# Comparison of MI-oriented versus CBT-oriented adjunctive treatments: impacts on therapeutic alliance and patient engagement during hospital treatment for an eating disorder

**DOI:** 10.1186/s40337-023-00818-8

**Published:** 2023-06-20

**Authors:** Jennifer S. Mills, Lauren E. Poulin, Gillian Kirsh

**Affiliations:** 1 Department of Psychology, York University, 4700 Keele St., Toronto, ON M3J 1P3 Canada; 2 Eating Disorders Program, North York General Hospital, 4001 Leslie St., North York, ON M2K 1E1 Canada

## Abstract

**Background:**

Our aim was to compare MI-oriented versus CBT-oriented adjunctive treatments to test whether an MI approach is superior in terms of improving therapeutic alliance and engagement among individuals with an eating disorder. The current study was a pilot randomized controlled trial with random allocation to either MI-oriented or a CBT-oriented adjunctive treatment group completed concurrently with a hospital-based group program for adults. Both adjunctive treatment conditions consisted of three individual therapy sessions and a self-help manual.

**Methods:**

Sixty-five outpatients receiving hospital treatment for a diagnosed eating disorder were randomly assigned to a treatment group. Measures of working therapeutic alliance, engagement, treatment completion, and clinical impairment were completed at preadmission, mid-treatment, and at the end of treatment.

**Results:**

Working alliance increased equivalently in both conditions over time in treatment. Similarly, there were no differences between conditions in terms of engagement. Regardless of therapy orientation, greater use of the self-help manual predicted lowered eating disorder risk; stronger patient ratings of therapeutic alliance predicted decreased feelings of both ineffectiveness and interpersonal problems.

**Conclusion:**

This pilot RCT provides further evidence that both alliance and engagement are important for treatment of an eating disorder; however, there was no clear advantage of MI over CBT as an adjunctive treatment approach to improving alliance or engagement.

*Trial registration*: ClinicalTrials.gov ID #NCT03643445 (proactive registration).

## Plain language summary

Drop-out is common among eating disorder treatment programs. Patients’ engagement in treatment and a positive relationship with their therapist may both be variables that are key to predicting treatment success. In this study we examined whether integrating MI-oriented individual therapy sessions plus a self-help manual would result in improved therapeutic alliance and/or engagement for adults attending a partial day hospital treatment program for an eating disorder as compared to similar CBT-oriented treatment. The MI-oriented treatment group was based on motivational interviewing principles that emphasize empathy, overcoming resistance to change, and individual’s autonomy over decisions that are in line with one’s values. The CBT-oriented treatment group was based on cognitive-behavioural principles that emphasize the need to change and the consequences of symptoms of an eating disorder. Patients’ ratings of the quality of their relationship with their therapist increased in both conditions over time. A stronger working alliance with one’s therapist was associated with lowered feelings of ineffectiveness and interpersonal problems, which are characteristics that are common among individuals with an eating disorder. Use of a self-help manual of either type showed some benefit at predicting reduced clinical impairment.

## Introduction

Eating disorders (EDs) seriously impact both mental and physical health, and are associated with high rates of mortality and relapse [1]. There is a considerable evidence base for cognitive behavioural therapy (CBT) for eating disorders with adults and older adolescents [2–4]. CBT reveals short and long-term remission rates of 37–69% [5]. A major complication in ED treatment is patients’ frequent reluctance to engage in treatment, resistance to change, and premature treatment drop-out [6, 7].

The therapeutic alliance is a collaborative relationship between client and therapist, where there is a strong bond and a shared understanding and agreement upon the goals and task of therapy [8]. It is considered to be a “common factor” and essential component among evidence-based psychotherapies [9]. Past research has found that early and mid-treatment therapeutic alliance is associated with improved outcomes in anorexia nervosa [10] and bulimia nervosa [11]. One study found that individuals with an ED who drop out of treatment prematurely rated the therapeutic alliance as significantly lower early in treatment than did patients who remained in treatment [12]. On the other hand, it has been suggested that therapists have an inflated sense of the importance of therapeutic alliance in eating disorder treatment outcomes [13]. The literature on the importance of therapeutic alliance in eating disorders is smaller compared to other psychological problems such as mood and anxiety disorders [14, 15].

Engagement in therapy, often measured as willingness and compliance in completing between-session tasks, is another psychotherapeutic process found to be an important component related to treatment outcome [16]. Homework compliance has been found to be negatively associated with dropout in cognitive and behavioural treatments [17, 18]. Thus, increased engagement in between-session tasks may be an indicator of improved motivation in eating disorder treatment, as well as increased likelihood of treatment completion.

Motivational Interviewing (MI) has been suggested for use with eating disorders because ambivalence toward change tends to be high [14, 19]. MI is a client- centered therapy which aims to enhance motivation to change through providing empathy, developing discrepancy, rolling with resistance, and supporting self-efficacy [20]. MI principles have been found to be positively associated with alliance and patient engagement [21]. Research reliably finds that baseline motivation predicts recovery from an ED (e.g.,[ 22]). Past research has found that adding MI as a pre-treatment to other therapies (e.g., CBT) leads to increased readiness to change, reduced psychological distress, and increased feelings of self-efficacy in ED patients, although these are generally small to moderate effects [7, 19]. ED patients randomly assigned to receive an MI pre-treatment have been found to have significantly higher completion rates of an intensive treatment program, compared to treatment as usual [23]. Previous research suggests that even self-help using a motivational style is a good option for patients with an ED [24]. Nevertheless, a 2013 review paper concluded ED symptoms have not reliably been shown to improve as a result of the addition of an MI pre-treatment [25]. Research investigating other psychological disorders (i.e., anxiety) has found that integrating MI into CBT treatment, rather than delivering MI before treatment, leads to improved homework compliance [16] as well as reduced resistance, increased engagement, and lasting reduction in symptomatology [26]. In other words, it may be beneficial to integrate key MI principles (e.g., empathy, rolling with resistance) into treatment of an ED, which may impact important psychotherapy factors such as therapeutic alliance and engagement.

## The current study

Psychotherapy researchers have suggested that focusing on the therapy process, rather than treatment modality alone, may be critical for increasing understanding the effective components of treatment [27]. The current study was a small, pilot randomized controlled trial that took place at an established partial-day hospital treatment program for adults with an ED. This trial compared two adjunctive treatments that took place concurrently with the standard group-based hospital treatment: a motivation-oriented group and a CBT-oriented (active control) group. Brief, adjunctive treatments can be successfully used concurrently with hospital-based group treatment for an ED [28].

The current study had four hypotheses. First, given the emphasis in MI on working alliance and the evidence base for improved alliance following MI treatments, it was hypothesized that the MI-oriented group would report greater improvements in working alliance over time in treatment versus the CBT-oriented group. Second, it was hypothesized that the MI-oriented group would report greater engagement in adjunctive treatment, as measured by use of the self-help manual, compared to the CBT-oriented group. Third, it was hypothesized that regardless of adjunctive therapy approach, higher therapeutic alliance and engagement would improve treatment completion. Fourth, it was predicted that higher therapeutic alliance and engagement would predict reduced clinical impairment at the end of treatment.

## Method

### Participants

The study was approved by the hospital Research Ethics Board and the Human Participants Review Committee at York University. Eligibility criteria included currently receiving treatment in the Adult Eating Disorders Program at North York General Hospital, being over 17 years old, having the ability to speak and read English, and being able to provide informed consent. Patients were 60 adults referred to the program from psychiatrists and physicians at the hospital and in the community. Patient demographics and the means and standard deviations for all measures are presented in Table 1. Psychiatric medication use was similar across the treatment conditions and included patients taking medication prescribed for bipolar disorder (MI: *n* = 2; CBT: *n* = 1), ADHD (MI: *n* = 4; CBT: *n* = 3), and depression/anxiety ((MI: *n* = 17, CBT: *n* = 19). There were no inclusion or exclusion criteria related to drug treatment. Patients received no other psychotherapy or physical therapy while participating in the study.

**Table 1:** Sample characteristics

Variables	Total sample (*N* = 65)	CBT-oriented group (*n* = 28)	MI-oriented group (*n* = 37)
Gender			
Female	59 (91%)	25 (89%)	34 (92%)
Male	4 (6%)	1 (4%)	3 (8%)
Other	2 (3%)	2 (7%)	0 (0%)
Mean age in years (*SD*)	30.80 (13.37)	30.19 (11.78)	31.24 (14.57)
Ethnic group			
White	39 (60%)	19 (68%)	20 (54%)
Asian	6 (9%)	3 (11%)	3 (8%)
Other	13 (20%)	4 (14%)	9 (24%)
Not reported	7 (11%)	2 (7%)	5 (14%)
Marital status			
Single	37 (57%)	16 (57%)	21 (57%)
Married	12 (18%)	3 (11%)	9 (24%)
Separated/divorced	7 (11%)	3 (11%)	4 (11%)
Common-law	4 (6%)	2 (7%)	2 (5%)
Not reported	5 (8%)	4 (14%)	1 (3%)
ED diagnosis			
Bulimia nervosa	30 (46%)	14 (50%)	16 (43%)
Anorexia nervosa	12 (18%)	4 (14%)	8 (22%)
Binge eating disorder	1 (2%)	0 (0%)	1 (3%)
ARFID	2 (3%)	1 (4%)	1 (3%)
EDNOS	19 (29%)	8 (29%)	11 (30%)
Not reported	1 (2%)	1 (4%)	0 (0%)
Previous ED treatment			
None	28 (43%)	13 (46%)	15 (41%)
Day hospital	17 (26%)	7 (25%)	10 (27%)
Inpatient	15 (23%)	5 (18%)	10 (27%)
Current program	2 (3%)	1 (4%)	1 (3%)
Other	1 (2%)	1 (4%)	0 (0%)
Not reported	2 (3%)	1 (4%)	1 (3%)

*ARFID* avoidant/restrictive food intake disorder, *EDNOS* eating disorder not otherwise specified. Percentages have been rounded to the nearest whole number and may not equal 100%

### Procedure

Patients provided written informed consent to participate in this research. Participants were offered the chance to participate as they were entering the partial hospital day treatment program. This program is a primarily CBT-based treatment group which runs for 3 evenings per week, from 3 p.m. to 8 p.m. At any time, 8–10 patients attend the day hospital program. It is an open group, accepting patients on an on-going basis. The average length of the treatment is 10–12 weeks. In addition to all of the standard group sessions, patients who chose to participate were randomly assigned to receive one of two adjunctive treatments: MI-oriented (*n* = 32) or CBT-oriented (*n* = 28) treatment. Adjunctive treatment included three individual meetings with a therapist occurring at the timepoints of preadmission (Time 1), 6-weeks into treatment (Time 2), and 10–12 weeks into treatment (Time 3). There was only one participant who attended the hospital program during data collection and did not wish to participate in the study. The leaders of the hospital program and study therapists were the same throughout the period of data collection, and the treatment forms and contents were consistent. Two of the hospital program therapists served as the MI therapists and one served as the CBT therapist. In other words, all participants had some exposure to CBT due to the existing partial day hospital program being CBT-based.

The MI-oriented group used MI principles to create a context for treatment which was patient-centered, non-judgemental, and which would aid patients in exploring their willingness to commit to behavioural changes required for recovery. This group received a self-help manual which included exercises for exploring and resolving ambivalence which they were encouraged to complete at their own pace and progress would be discussed together. This adjunctive treatment was delivered by two experienced staff members in the ED hospital program who had expressed interest in being trained in Motivational Interviewing. Staff members were trained in MI treatment and principles through a 16-h “Advanced Motivational Interviewing” workshop, led by Dr. William Miller, one of the founders of MI. This workshop included demonstrations and opportunities for experiential skill practice.

The CBT-oriented group was intended to be an active control group to control for possible effects of therapist interaction and any form of self-help. The principles implemented in this group were intended to be similar to treatment-as-usual in the current and other ED hospital programs. Content covered in the individual therapy sessions and self-help manual included teaching patients about the causes of eating disorders, obstacles to recovery, and the importance of behavioural changes required for symptom interruption and recovery. Similar to the MI-oriented group, patients were encouraged to complete the manual at their own pace and progress would be discussed together. This adjunctive treatment was delivered by an experienced staff member in the ED hospital program. To control for expertise and professional development, this staff member completed a CBT workshop equivalent in length to the MI workshop, led by Dr. Christine Padesky, expert trainer in CBT. Patients in both treatment conditions received the same care in terms of their hospital treatment and interactions with staff, with the only difference between them being the orientation of their individual therapy meetings and self-help manual.

In summary, this was a single-factor randomized controlled design; participants all attended a partial day hospital program and were randomly assigned to an adjunctive treatment condition which followed either a CBT or MI orientation (receiving both individual treatment and a self-help manual).

### Measures

At each individual meeting (i.e., at each of the 3 time points), patients completed self-report questionnaires that included: the Working Alliance Inventory (WAI-SR; [29]), self-help manual use, and the Eating Disorder Inventory (EDI-3; [30, 31]).

#### Working alliance inventory—short revised (WAI-SR)

The WAI-SR [29] is a commonly used self-report measure for assessing the collaborative and affective bond between therapist and patient, and is consistently related to therapeutic outcome [32]. It is a 12-item measure containing three subscales measuring different aspects of the alliance. These subscales include Bond (strength of relational bond between patient and therapist), Task (agreement on therapeutic tasks), and Goal (agreement on therapeutic goals). Each item is assessed on a scale from 1 (Strongly Disagree) to 5 (Strongly Agree). Higher scores reflect better alliance. The WAI-SR has good reliability and convergent validity, and good fit with the proposed Bond-Task-Goal model [29, 33, 34]. Internal consistency coefficients (alpha) at Time 1 were good and were as follows: Bond = 0.786; Task = 0.780; Goals = 0.822.

#### Self help manual use

A two-item measure was created for this study assessing how much time the patient had spent reading the self-help manual, and how much of the self-help manual the patient read. Both manuals were the same length and contained text, worksheets, and figures. Without doing any of the exercises, we estimated that the average time it would take an adult to simply read the manual from start to finish would be 3–5 h. Time spent re-reading any portions, thinking about suggested exercises, and completing the worksheets could take many more hours. Time spent reading the manual was measured on a Likert-type scale of 1–5 (1 = less than one hour, 2 = 1–5 h, 3 = 5–10 h, 4 = 10–15 h, and 5 = 15 + hours). Amount of manual read was also measured on a Likert-type scale (1 = none of the manual, 2 = less than half of the manual, 3 = half of the manual, 4 = more than half of the manual, and 5 = the entire manual). This questionnaire was administered at each individual meeting. Because questions asked about self-help manual use *since the manual was received*, data from the final meeting were used as a measure of total self- help manual use. When data were missing at the final meeting, the most recent available time point was used. This measure was used as a proxy measure for patient engagement in treatment between-sessions since patients were encouraged to use the manual as a part of their treatment.

#### Eating disorders inventory—3 (EDI-3)

The EDI-3 [30] is a self-report questionnaire assessing eating disorder symptoms and psychological features. The EDI-3 is commonly used worldwide in both research and clinical work. It has shown good discriminative validity and excellent sensitivity, specificity, and reliability [25, 30, 35]. The current study used the six EDI-3 composite scores representing clinical impairment: Eating Disorder Risk, Ineffectiveness, Interpersonal Problems, Affective Problems, Overcontrol, and General Psychological Maladjustment [30].

### Data analytic plan

R Studio was used for all statistical procedures. To examine changes over the course of treatment, analyses were performed using a mixed-effects regression model (also known as a random effects regression model). This type of model is ideal for analyzing longitudinal data, as participants with missing data across time are still included in the analysis and all available data can be used [36]. In addition, change is estimated for each participant, rather than looking at average trends for the group. Time is included as a random effect to allow for nesting of time points within participants.

Prior to running the main analyses, potential issues with normality were examined among the dependent variables. Most variables were negatively skewed; however, a mixed-effects regression model does not require normality among variables, but rather a normality in the residuals of the given model. Because the transformation of variables makes unstandardized effect sizes more difficult to interpret, we chose to not transform these variables. However, to ensure that results were not driven by this choice, all analyses were run with and without variable transformation. Results were largely consistent regardless of the strategy used. Any exceptions are reported below.

## Results

### Descriptive statistics

Differences between the two treatment conditions on demographics were assessed using t-tests for continuous variables, and chi square analyses for categorical variables. When the expected value for a categorical variable was less than 5, Fisher’s exact test was used. There were no significant differences between treatment conditions on gender, age, ethnic group, marital status, ED diagnosis, or previous treatment. Participant characteristics are shown in Table 1.

### Changes in working alliance over time as a function of group

T-tests and chi- square analyses indicated no difference between conditions on WAI-SR score, self-help manual use, or treatment dropout. A mixed-effects linear regression model was first performed to examine the changes in WAI over time across all participants. Treatment group was then included in the model to ascertain whether the different conditions showed different WAI trajectories. There was a significant effect of time on total WAI score across all participants, such that total WAI score increased by 0.26 points at each measurement (*β* = 0.26, *p* < .001). When treatment group and treatment group by time interaction variables were added to the model, these variables showed no significant effects. Thus, total WAI score increased over time in both the CBT-oriented and the MI-oriented treatment conditions.

Total working alliance was separated into its components to examine whether a specific component of the working alliance was driving this overall increase. Again, models were run with time as a predictor of WAI, then including treatment group and an interaction term. Results demonstrated a significant effect of time on each subscale of the WAI. Bond increased by an average of 0.16 points at each measurement (*β* = 0.16, *p* < .05); Task Agreement increased by an average of 0.15 points at each measurement (*β* = 0.15, *p* < .05); Goal Agreement increased an average of 0.46 points at each measurement (*β* = 0.46, *p* < .001). Thus, all components of WAI saw an increase over treatment, with goal agreement showing the largest increase. Treatment group was not a significant predictor of WAI in any of the three models. An interaction between time and treatment group was not observed for either Bond or Goal Agreement, but the interaction term was nearing significance for predicting WAI Task Agreement score (*β* = − 0.27, *p* = .058). Specifically, for the CBT-oriented group there was a 0.29 point average increase in task agreement at each time point. For those in the MI-oriented group, there was only a 0.02 average increase in task agreement at each time point, indicating that task agreement remained stable over time. Although this did not reach statistical significance, when WAI variables were transformed to correct for skewness, the interaction was significant (*p* = .03). Results are shown in Table 2.

**Table 2:** Measures of interest as a function of adjunctive treatment group

Measure	Total sample (*N* = 65)	CBT-oriented group (*n* = 28)	MI-oriented group (*n* = 37)
WAI-SR			
Time 1 (*n* = 60)	*M* = 3.8, *SD* = 0.81	*M* = 3.72 *SD* = 0.79	*M* = 3.86 *SD* = 0.84
Time 2 (*n* = 50)	*M* = 4.12 *SD* = 0.61	*M* = 4.27 *SD* = 0.54	*M* = 4.01 *SD* = 0.65
Time 3 (*n* = 36)	*M* = 4.38 *SD* = 0.65	*M* = 4.33 *SD* = 0.88	*M* = 4.43 *SD* = 0.41
WAI bond			
Time 1 (*n* = 60)	*M* = 4.13 *SD* = 0.83	*M* = 4.07 *SD* = 0.78	*M* = 4.18 *SD* = 0.89
Time 2 (*n* = 50)	*M* = 4.39 *SD* = 0.69	*M* = 4.52 *SD* = 0.58	*M* = 4.29 *SD* = 0.75
Time 3 (*n* = 36)	*M* = 4.53 *SD* = 0.73	*M* = 4.39 *SD* = 0.97	*M* = 4.64 *SD* = 0.46
WAI task			
Time 1 (*n* = 60)	*M* = 3.54 *SD* = 0.95	*M* = 3.43 *SD* = 0.91	*M* = 3.65 *SD* = 0.98
Time 2 (*n* = 50)	*M* = 3.58 *SD* = 0.83	*M* = 3.74 *SD* = 0.85	*M* = 3.47 *SD* = 0.80
Time 3 (*n* = 36)	*M* = 4.04 *SD* = 0.85	*M* = 4.14 *SD* = 0.97	*M* = 3.96 *SD* = 0.75
WAI goals			
Time 1 (*n* = 59)	*M* = 3.70 *SD* = 1.09	*M* = 3.66 *SD* = 1.12	*M* = 3.73 *SD* = 1.09
Time 2 (*n* = 50)	*M* = 4.38 *SD* = 0.68	*M* = 4.54 *SD* = 0.50	*M* = 4.27 *SD* = 0.77
Time 3 (*n* = 36)	*M hasis> = 4.58 x* *SD* = 0.66	*M* = 4.45 *SD* = 0.89	*M* = 4.69 *SD* = 0.39
Time reading manual	*M* = 1.69 *SD* = 0.93	*M* = 1.73 *SD* = 0.88	*M* = 1.66 *SD* = 0.97
Amount of manual read	*M* = 2.19 *SD* = 0.91	*M* = 2.23 *SD* = 0.87	*M* = 2.16 *SD* = 0.95
Treatment completion			
No	26 (40%)	11 (39%)	15 (41%)
Yes	39 (60%)	17 (61%)	22 (59%)

Percentages have been rounded to the nearest whole number

### Differences in self-help manual use and treatment completion between conditions

T-tests were performed to examine differences in self-help manual use between treatment conditions. There was no difference in total time spent reading the manual, both conditions reading about one hour (psychoeducation-oriented: *M* = 1.73, *SD* = 0.88; motivation-oriented *M* = 1.66, *SD* = 0.97; *p* = .62). There was no difference in total amount of manual read, with both conditions reading less than half the manual (CBT-oriented: *M* = 2.23, *SD* = 0.87; MI-oriented: *M* = 2.16, *SD* = 0.95; *p* = .61). Chi-square analyses indicated no significant difference between conditions on treatment dropout.

### Impact of working alliance and self-help manual use on treatment completion and clinical impairment

#### Treatment completion

For this outcome measure of interest only, a mixed effects logistic regression was performed due to the binary nature of the outcome variable. Total WAI-SR score and total time spent reading the manual were entered as predictors of completion, while controlling for random effects of time grouped within-subject to account for the longitudinal nature of the data. There was no significant effect of total WAI score (odds ratio = 2.58, *p* = .57) or time spent reading the manual (odds ratio = 1.49, *p* = .84) on whether participants completed treatment, though the odds ratios were relatively large.

#### Clinical impairment

Mixed effects linear regression models were performed to examine whether working alliance (WAI-SR total score) and time spent reading the self-help manual predicted clinical impairment, as measured by the six composite scales of the EDI-3. Patient T scores on EDI scales were used to facilitate interpretation of results (10T represents 1 standard deviation).

##### Influence of self-help manual use on clinical impairment

The effect of self-help manual use on eating disorder risk was nearing significance, such that for every unit increase of in self-help manual use, eating disorder risk decreased by 2.64T (*β* = − 2.62, *p* = .054). In other words, increasing self-help manual reading by 5 h was associated with about ¼ of a standard deviation decrease in Eating Disorder Risk. There were no significant effects of self-help manual use on Ineffectiveness, Interpersonal Problems, Affective Problems, Overcontrol, or General Psychological Maladjustment (all *p*s > 0.06).

##### Influence of working alliance on clinical impairment

There was a significant effect of WAI on Ineffectiveness, such that a 1 unit increase in total WAI predicted a 2.77T decrease in Ineffectiveness (*β* = − 2.77, *p* = .03). There was also a significant effect of WAI on Interpersonal Problems, such that a one unit increase in total WAI predicted a 3.29T decrease in Interpersonal Problems (*β* = − 3.29, *p* = .01). WAI score did not significantly predict Eating Disorder Risk, Affective Problems, Overcontrol, or General Psychological Maladjustment (all *p*s > 0.18).

## Discussion

This study was a preliminary investigation in the form of a pilot randomized controlled trial of the impact of implementing an MI-oriented adjunctive treatment into a partial day hospital program for adults with an ED. The MI-oriented group was compared to an active control group that was CBT-oriented. Both adjunctive treatment conditions received three sessions of individual therapy plus a self-help manual. Contrary to our first and second hypotheses, the results showed that the two treatment conditions were equivalent on increases in therapeutic working alliance during time in treatment and on engagement as measured by self-help manual use. The results did not confirm our third hypothesis that alliance and engagement would positively predict treatment completion. Consistent with our fourth hypothesis, regardless of adjunctive treatment modality, working alliance and engagement were predictive of a reduction in various aspects of clinical impairment.

Participants’ ratings of their working therapeutic alliance with their individual therapist improved over time in treatment in both treatment conditions, with the *goal agreement* subscale of the working alliance measure showing the greatest increase over time. Improvement in alliance over the course of treatment is consistent with past research (e.g., [37, 38]). When comparing other aspects of alliance between the two treatment conditions, the only significant difference observed was in the progression of *task agreement*, which improved over time in the CBT-oriented group, while it generally remained the same in the MI-oriented group. Although not consistent with our hypothesis that MI would have an advantage over CBT in terms of alliance, this result may be because the CBT-oriented sessions and self-help manual provided information about the importance of change and specific strategies for how to change. In contrast, MI emphasizes helping patients to come to their own decisions about their treatment [39].

Another aim of the study was to examine engagement in treatment since this is often cited as a barrier to ED recovery. Patients’ level of engagement was operationalized through their use of the self-help manual provided to them. Both conditions were given a manual that they were encouraged, but not required, to read. The results showed that neither group used the self-help manual to a large extent, with most participants reading the manual for less than 5 h in total and reading fewer than half the chapters. Other research has found that brief Motivation Enhancement Therapy did not result in increased use of a self-help manual compared to simply providing a self-help manual [40]. Furthermore, the hospital setting of the current study already provides a lot of written materials to patients. It could be that participants felt that the self-help manual given to them was too much extra reading. Study replication in a different setting and where less printed material is routinely provided might reveal different results, but MI showed no advantage over CBT in the current study in terms of use of a self-help manual.

There are several possible therapy-related explanations for why the hypothesized benefits of MI-oriented adjunctive treatment on both alliance and engagement did not emerge. First, there were only three individual therapy sessions. Differences between the MI-oriented versus CBT-oriented treatments may have been diluted by the fact that the majority of patients’ total treatment was the same between these two conditions. Furthermore, CBT does improve motivation to recover from an ED [41]; it may do as good a job as MI depending on various factors. Therapeutic alliance and/or treatment engagement are likely influenced by individual differences among patients (e.g., personality). Patients who read more of the manual may have done so because they are inherently more conscientious or were worried about upsetting their individual therapist if they did not read it. In addition, working alliance was measured by self-report and there can be a response bias due to patient attempts at impression management [42]. Moreover, therapist effects exert an influence on treatment and are difficult to control. A close, empathetic alliance with a therapist can be formed regardless of treatment modality [43]. All therapists in the current study were highly trained and experienced therapists. Finally, MI sometimes demonstrates a ‘sleeper effect,’ with benefits appearing up to 15 months post-treatment [26, 44, 45]. Past research has indicated that changes in self-efficacy resulting from MI seems to persist, even when therapy skills have been forgotten. Unfortunately, follow-up data are not available due to program interruptions related to the COVID-19 pandemic.

### Influence of alliance and engagement on treatment outcome and clinical impairment

Contrary to our third hypothesis, we found that therapeutic working alliance scores and self-help manual use did not significantly predict treatment completion. Results revealed that a 1 unit increase in WAI-SR total score increased the odds of treatment completion by 2.5, and a 1 unit (i.e., 5 h) increase in time spent reading the manual increased the odds of completion by 1.49. In other words, despite not reaching statistical significance, based on the demonstrated odds ratios it can be extrapolated that increasing alliance and engagement between sessions is not detrimental and, if anything, results in improved treatment completion rates. This is consistent with previous research [13, 14]. Future research should use a larger sample size and multiple measures of between-session engagement to determine whether there is a clinically meaningful and reliable effect.

Finally, in partial support of our fourth hypothesis, working alliance and self-help manual use did predict some aspects of ED clinical impairment across treatment conditions. First, increased self-help manual use predicted decreased *eating disorder risk*, conceptualized as the cardinal component of disordered eating [25, 26]. A reduction in clinical impairment is a major goal of treatment, and the fact that self-help manual use predicted reduced levels of symptomatology is an important takeaway from the current study. Although few individuals used the manual in its entirety, those who used more of the manual had a significantly reduced risk for ongoing eating disorder symptoms. Although self-help manual use may not be an ideal measure of treatment engagement, this measure tapped into something that is related to clinical improvement. Self-help manual use may be related to another predictor, such as conscientiousness, treatment expectations, or other predictors of positive outcome. Qualitative survey methods may be helpful in future studies to examine self-help manual use (and non-use), and engagement in treatment more broadly [46].

Higher patient ratings of working alliance with their therapist predicted decreased *ineffectiveness*, which includes feelings of general inadequacy, worthlessness, and not being in control of one’s life. A positive relationship with one’s therapist contributes to patients feel cared for and valued, which, in turn, may lead to feelings of greater self-worth control over one’s life. Higher patient ratings of therapeutic working alliance also predicted decreased *interpersonal problems*. A strong therapeutic bond may model a personal relationship that feels safe and supportive, thereby building positive experiences that may be carried into other relationships. In the current study, neither working alliance nor self-help manual use showed any relation to other aspects of clinical impairment, such as affective problems (made up of the components of interoceptive deficits and emotion dysregulation), overcontrol (made up of the components of perfectionism and asceticism), or general psychological maladjustment.

### Clinical implications

This research offers some tentative clinical implications for the treatment of eating disorders among adults in intensive hospital treatment, despite being a smaller scale study. The findings clearly show that a positive alliance with a therapist helps in the reduction of eating disorder symptoms. Care must be taken by therapists in developing a bond with clients and ensuring agreement on tasks and goals throughout treatment. Alliance tends to increase over time, so therapists should not be overly concerned if an alliance takes some time to build.

However, if alliance is not improving over treatment or alliance is consistently poor, it may be worth transitioning a patient to a therapist who may be a better fit, if such resources are available.

The finding that self-help manual use was associated with reduced eating disorder risk may indicate a place where therapists may need to pay special attention. Extra care may be taken to check in on patients who report not engaging in any between-session activities, sensitively exploring their reluctance to do so. As they are not engaging outside of treatment, care may be taken within group treatment to engage them in learning and practicing skills. Those patients who show much higher motivation to engage in activities between sessions may need less focus in group sessions.

## Limitations and future direction

This study was limited by the small sample size. Like many hospital ED treatment programs, only a small number of patients are enrolled at any time. There was no a priori sample size estimation and data collection was disrupted by the COVID-19 pandemic. Because of the small sample size, we did not correct for multiple comparisons. Although study therapists attended equivalent professional development sessions prior to the start of the study, there was no recording of individual sessions or fidelity coding due to patient confidentiality and ethical constraints. Program staff chose the modality to which they were assigned, in order to control for treatment loyalty and therapist preference, but it is possible that this introduced a confound of therapist self-selection. The current findings should be interpreted cautiously and future research should aim to recruit a larger sample size and at multiple sites. We did not direct participants to read the self-help manuals. The homework compliance measure we used may have been insufficiently valid and unable to predict the outcomes. As mentioned above, despite our attempts to match the manuals in terms of readability, length, and appearance, factors other than treatment orientation could have influenced how much of their manual participant reported reading. We did not include a control group that received neither MI-oriented individual therapy not CBT-oriented individual therapy, which could have informed whether there is a synergistic effect of the adjunct interventions. Qualitative data should also be gathered from patients in the future, examining their reasoning for manual use and their satisfaction with the treatment. Participants in the CBT-oriented group received a stronger or more “potent” dose of that theoretical approach owing to the fact that much of the day hospital program was based on CBT principles. We could not change the day hospital curriculum and chose an established comparison group as an active control. Finally, effects should be further examined at a longer-term follow-up meeting (e.g., 6 months post discharge) to determine whether there are any sleeper effects of the MI-oriented treatment, as mentioned above. Furthermore, other researchers have conducted qualitative research that suggests a number of potentially malleable factors that may affect ED recovery motivation (e.g., removing triggers, focusing on obligation to others, getting involved in meaningful causes, securing non-judgmental support, building hope for the future) and that could be specific targets in motivational interventions for an ED.

## Conclusions

We compared two conditions in a small randomized controlled trial of an MI-oriented adjunctive treatment and a CBT-oriented adjunctive treatment, consisting of individual therapy sessions and a self-help manual, among adults who were attending a partial day hospital treatment program for an ED. We found no significant differences between the MI-oriented group and the CBT-oriented group on increases in therapeutic alliance and engagement over treatment. In terms of psychotherapy process predictors, improved alliance and engagement were associated with better clinical outcomes on critical components of eating pathology across conditions at the end of hospital treatment, thus highlighting the importance of finding ways to target and improve these specific psychotherapy processes across ED treatment approaches. In conclusion, alliance and engagement matter for patients in a hospital-based treatment, but there is no clear winner in terms of an MI versus CBT-oriented adjunctive treatment.

## Data Availability

The data that support these findings are not publicly available due to privacy or ethical restrictions. Self-help manuals are available from the corresponding author upon request.
